# Supplementary figures and images for: Using an ideal observer analysis to investigate the visual perceptual efficiency of individuals with a history of non-suicidal self-injury when identifying emotional expressions
Source: PLoS One. 2020 Feb 3;15(2):e0227019. doi: 10.1371/journal.pone.0227019 (PMC6996801; doi:10.1371/journal.pone.0227019)

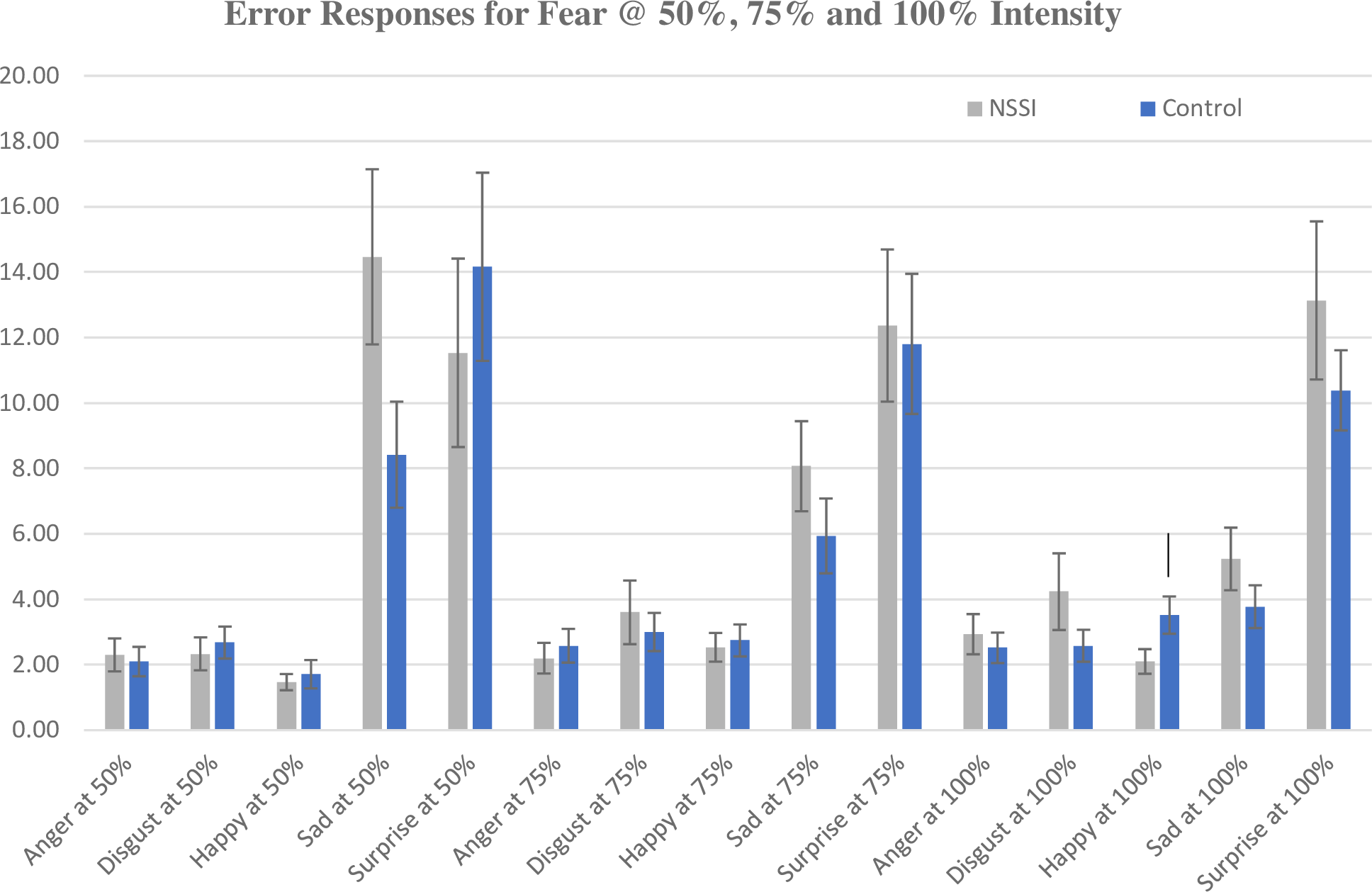

Supplement: S1 Fig — (TIF) [file pone.0227019.s001.tif]

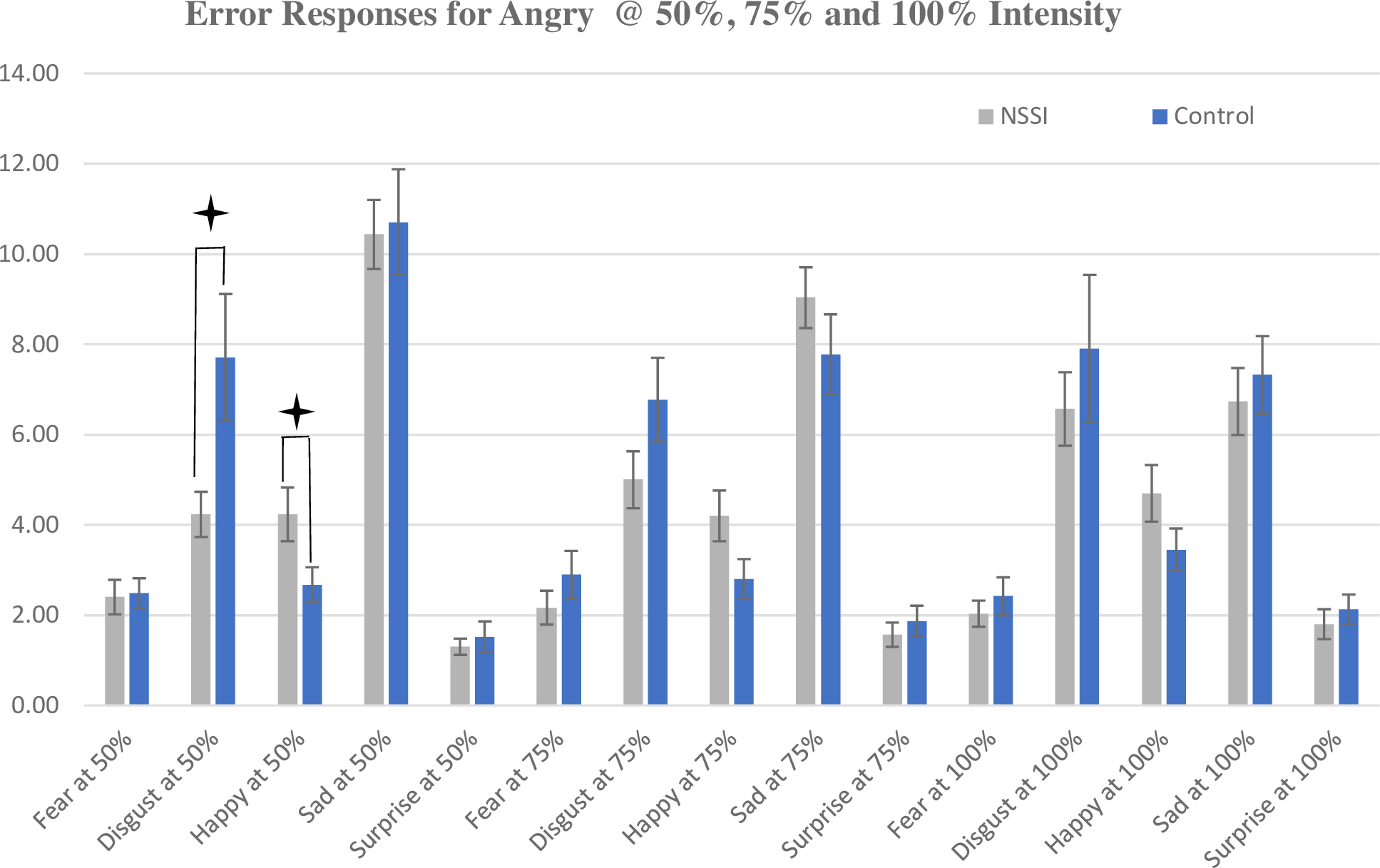

Supplement: S2 Fig — (TIF) [file pone.0227019.s002.tif]

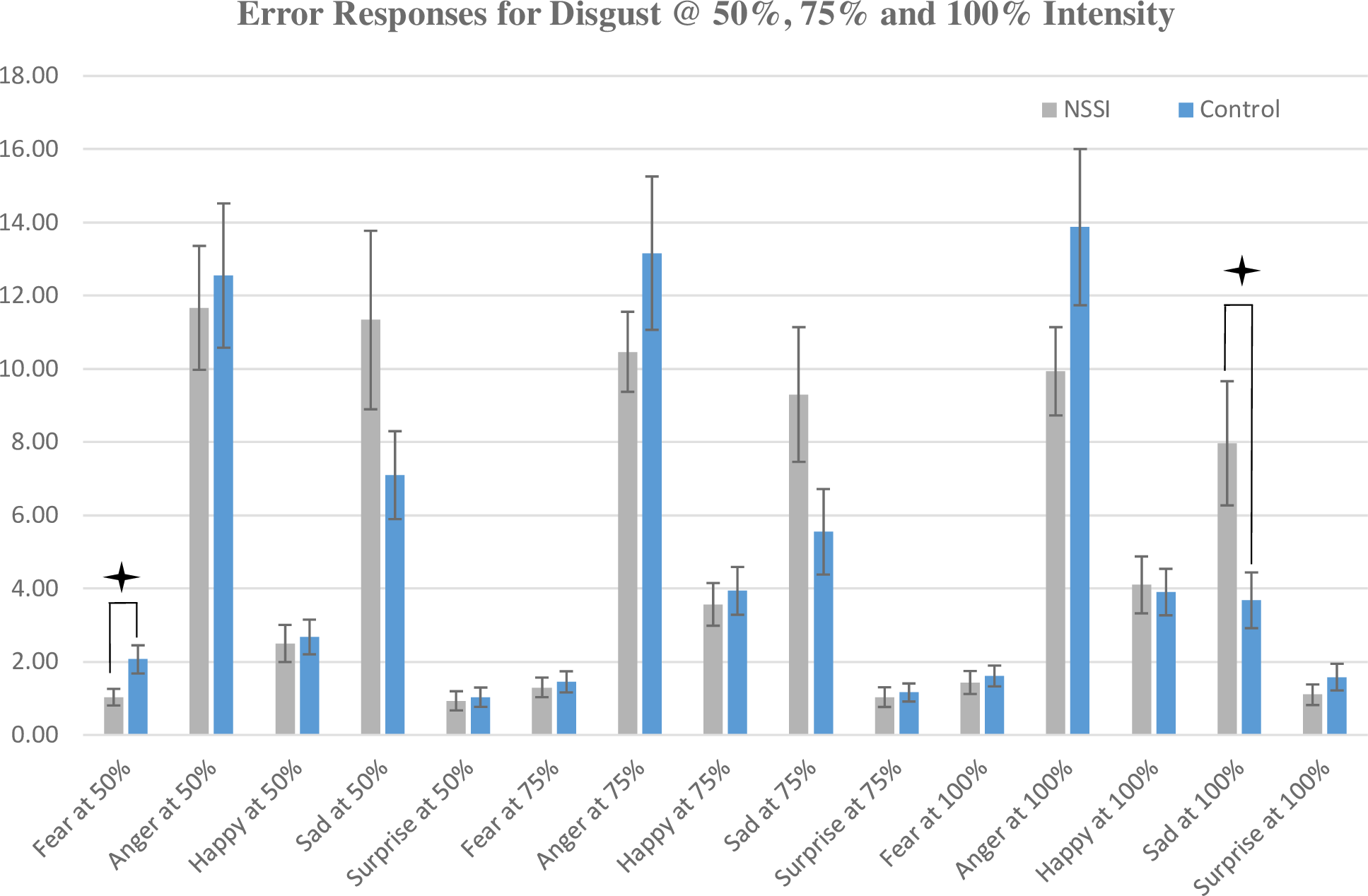

Supplement: S3 Fig — (TIF) [file pone.0227019.s003.tif]

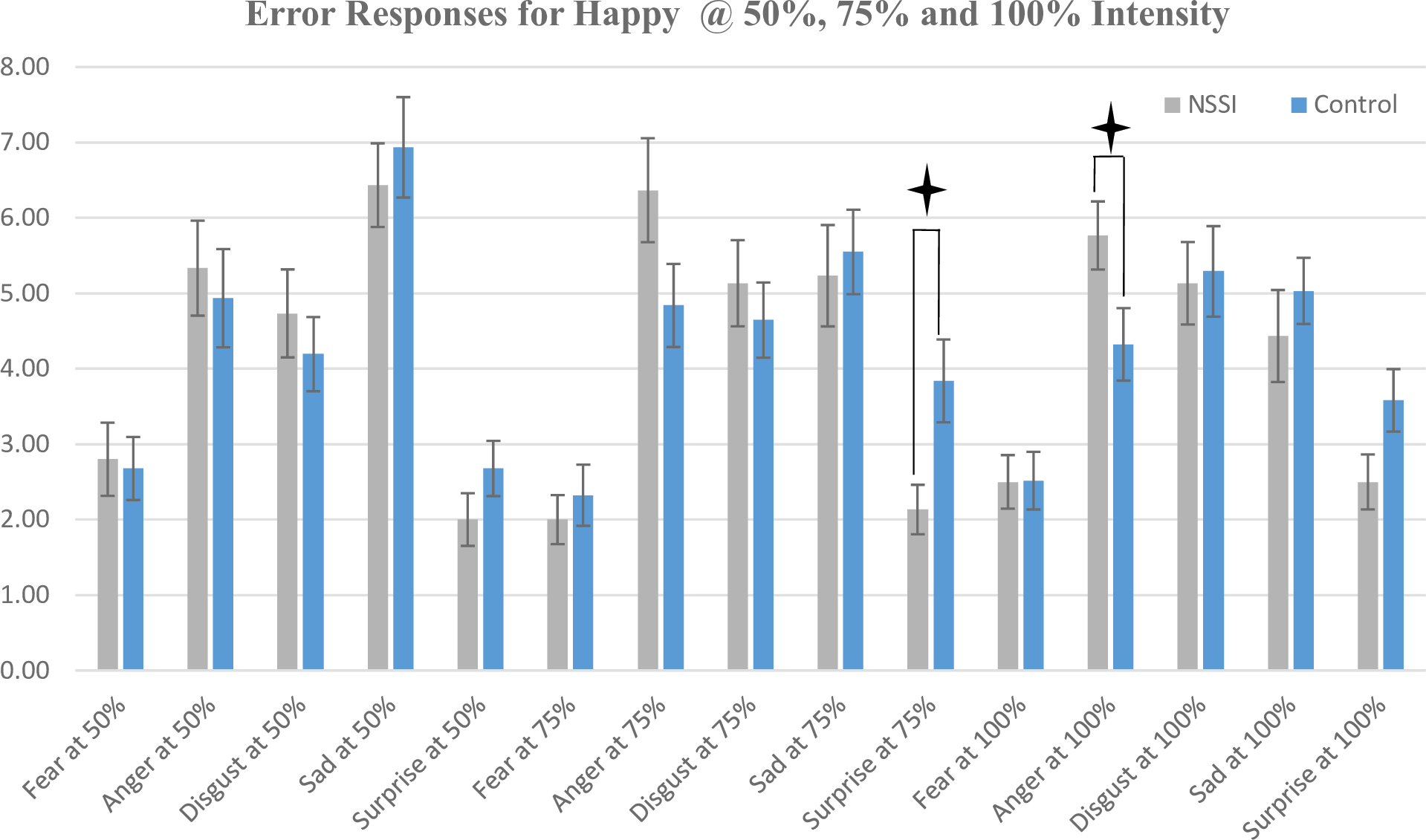

Supplement: S4 Fig — (TIF) [file pone.0227019.s004.tif]

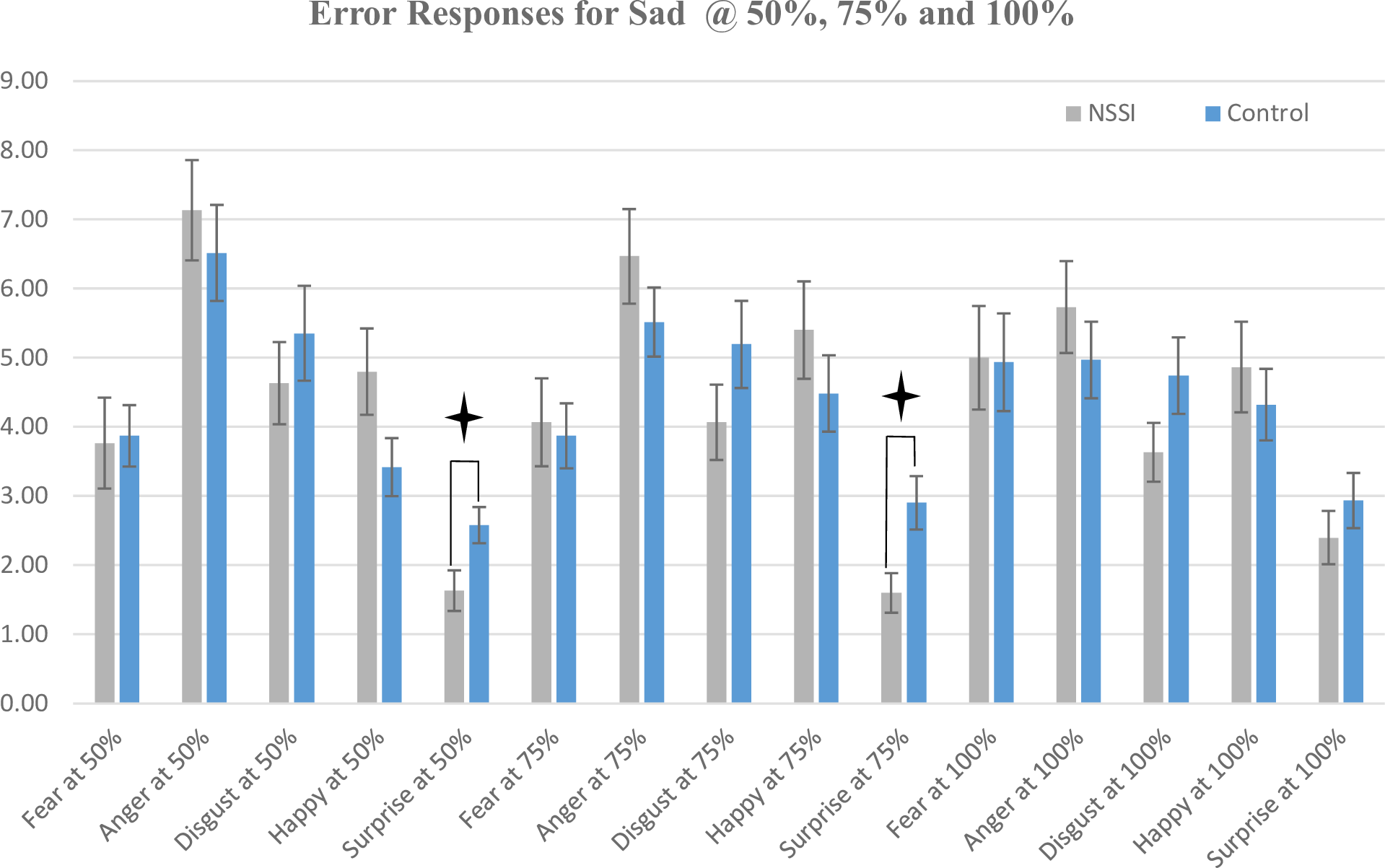

Supplement: S5 Fig — (TIF) [file pone.0227019.s005.tif]

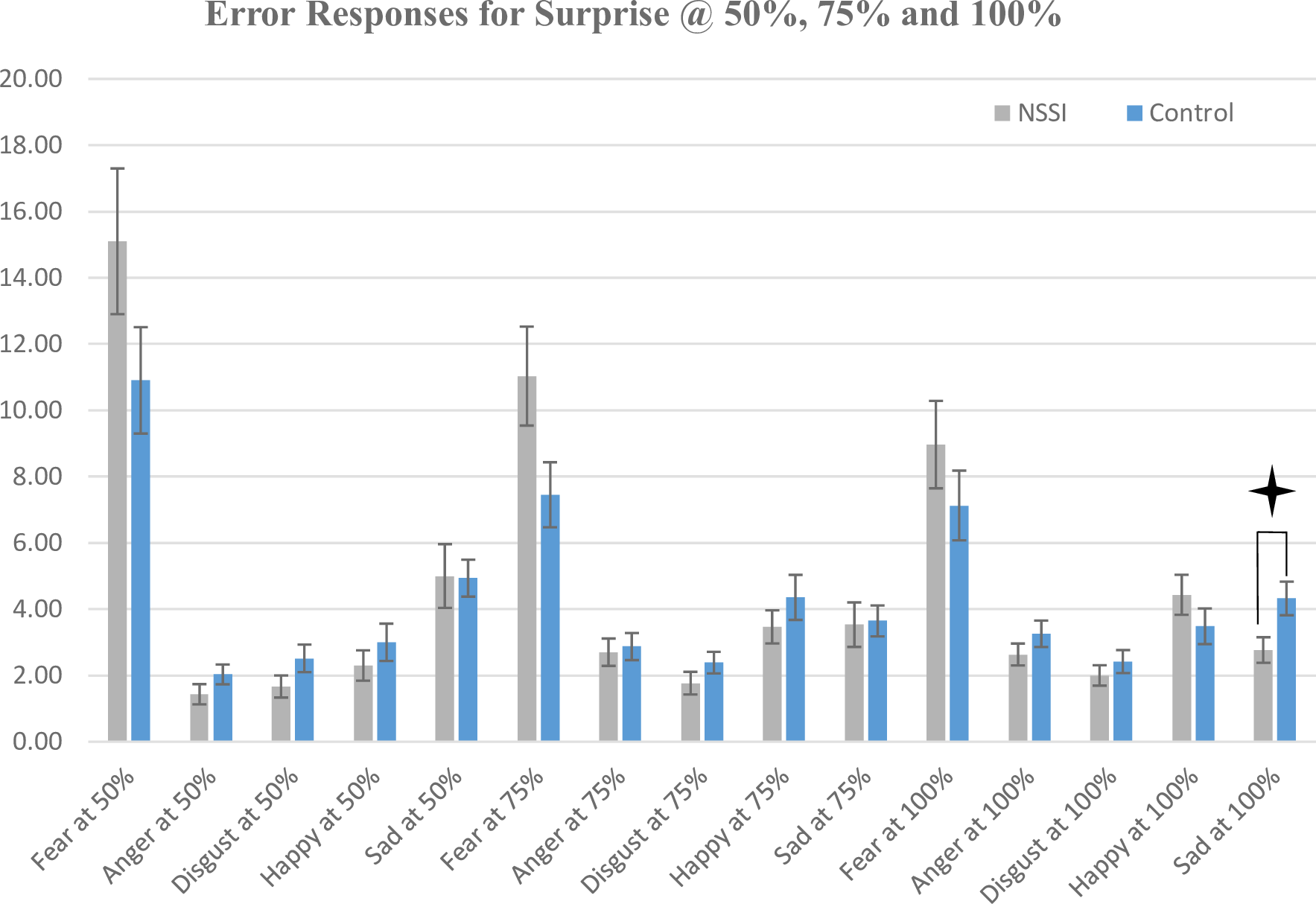

Supplement: S6 Fig — (TIF) [file pone.0227019.s006.tif]

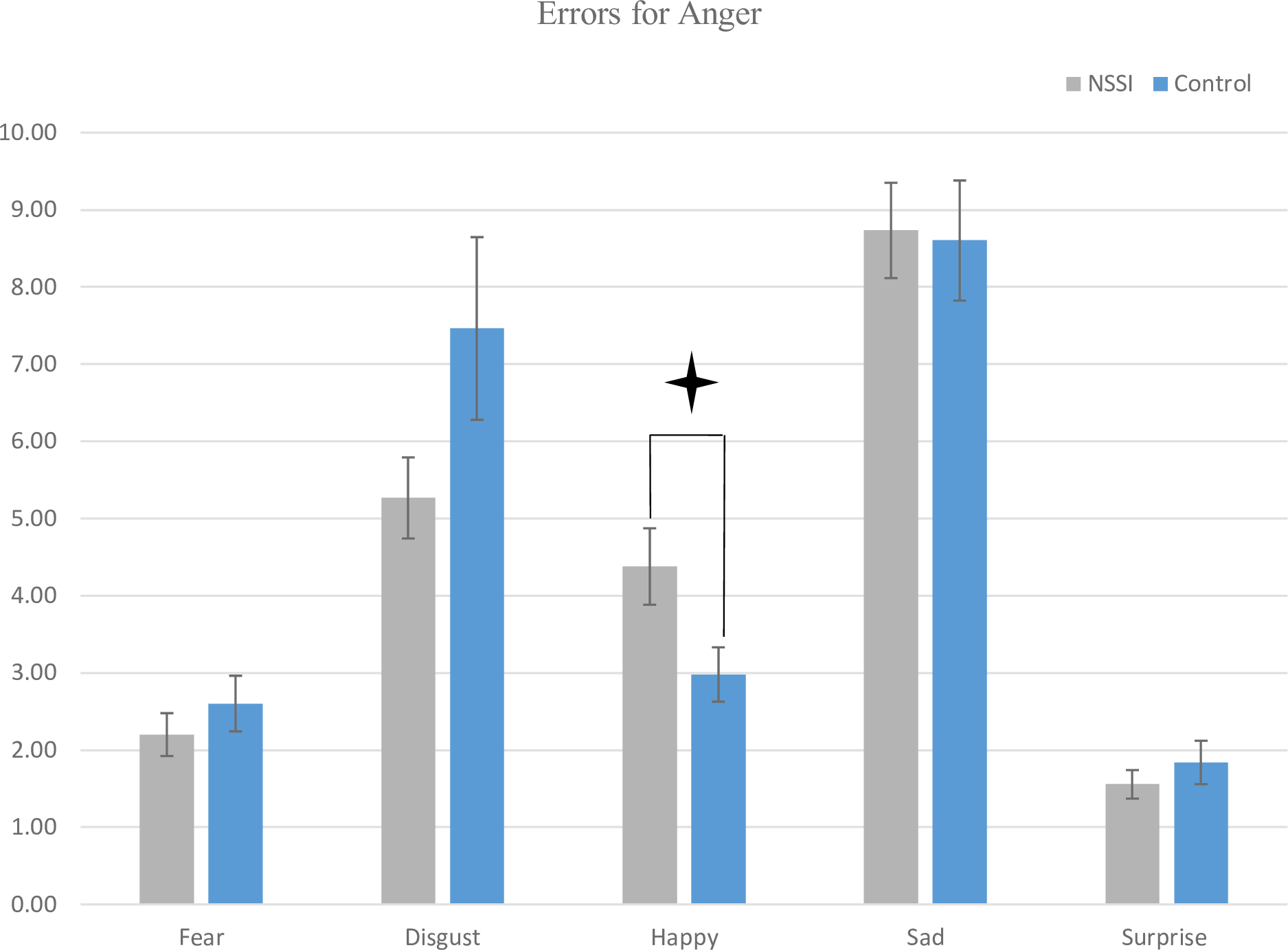

Supplement: S7 Fig — (TIF) [file pone.0227019.s007.tif]

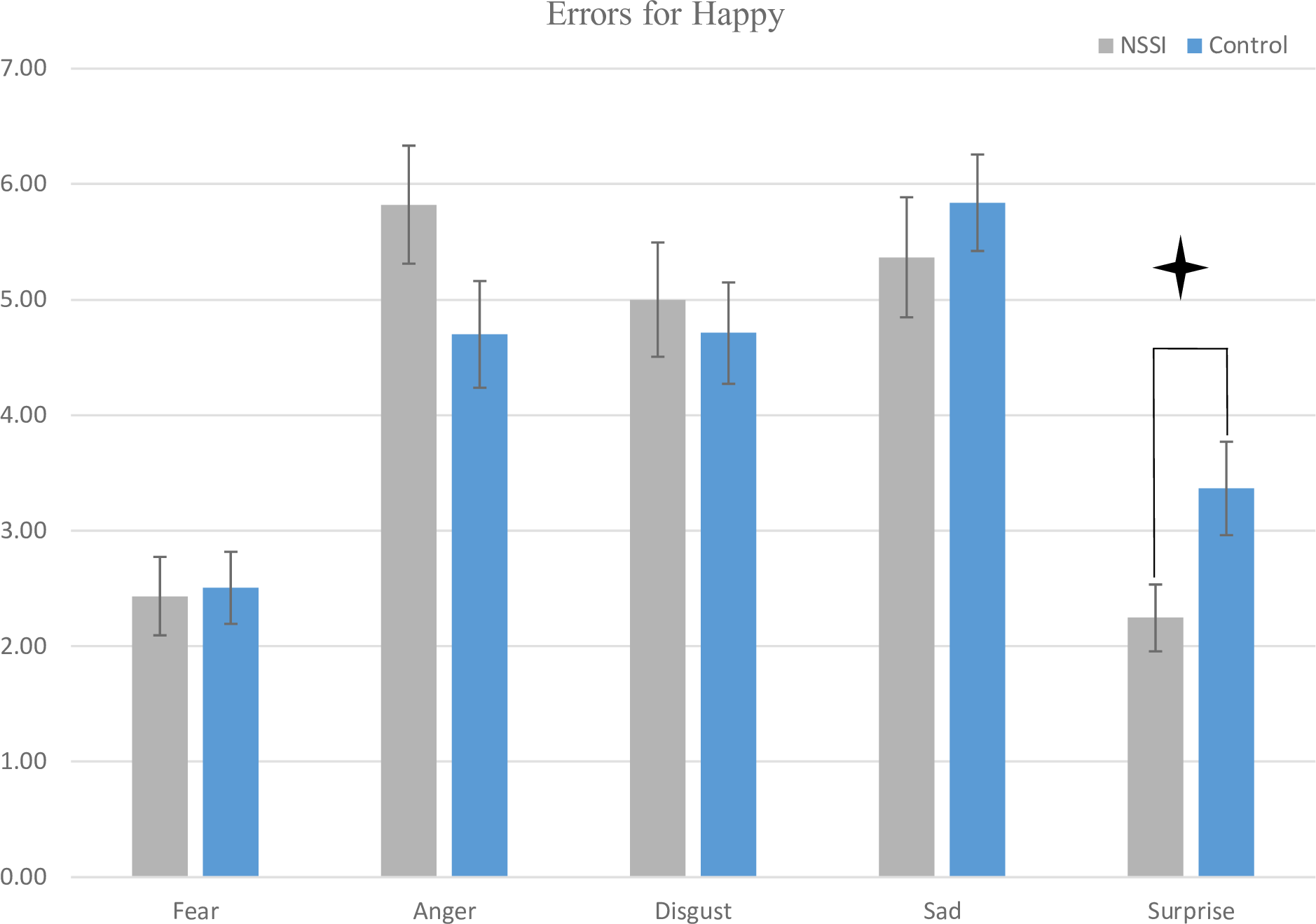

Supplement: S8 Fig — (TIF) [file pone.0227019.s008.tif]

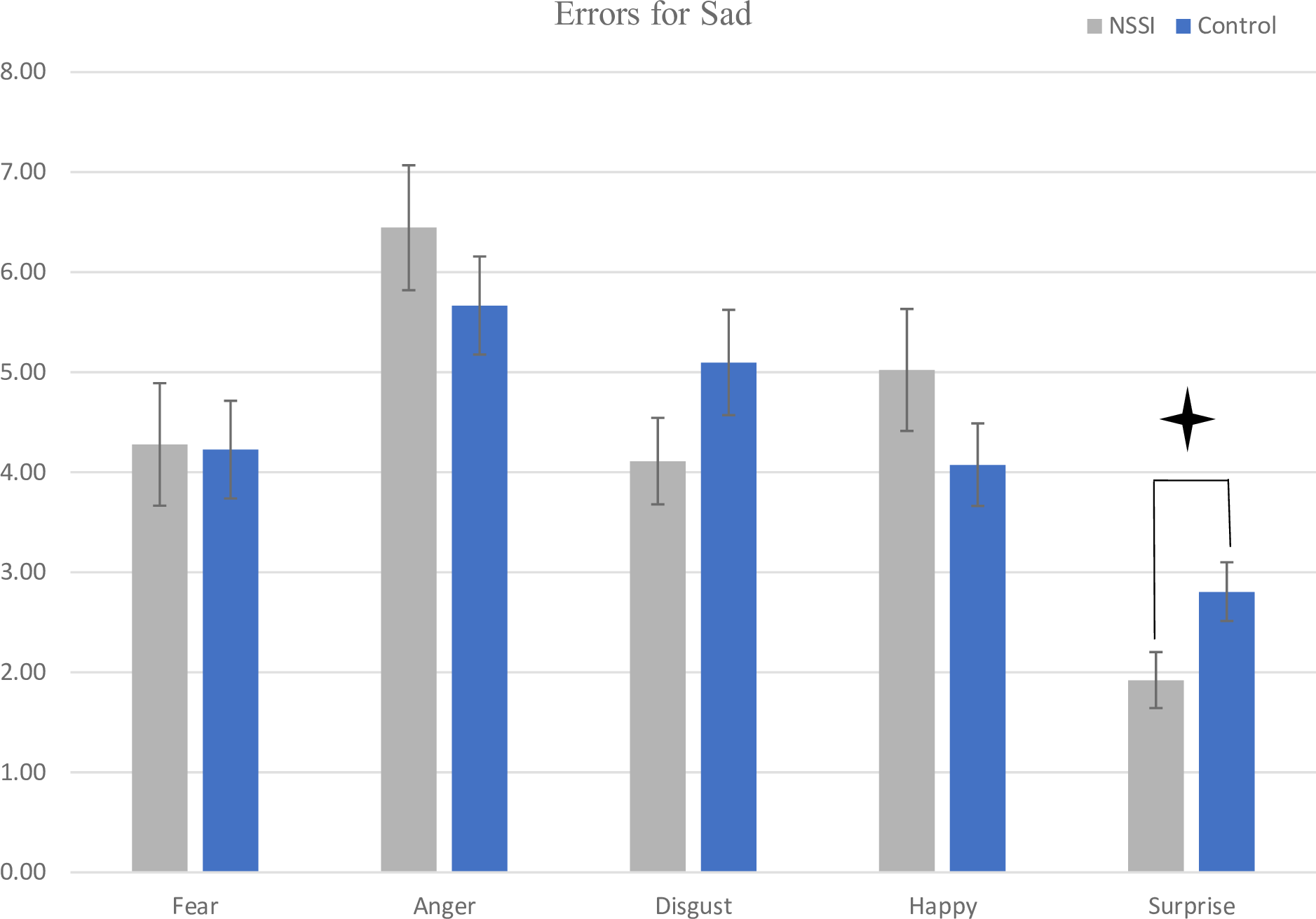

Supplement: S9 Fig — (TIF) [file pone.0227019.s009.tif]
