# Supplementary material for: Using an ideal observer analysis to investigate the visual perceptual efficiency of individuals with a history of non-suicidal self-injury when identifying emotional expressions
Source: PLoS One. 2020 Feb 3;15(2):e0227019. doi: 10.1371/journal.pone.0227019 (PMC6996801; doi:10.1371/journal.pone.0227019)
Supplement: S1 File — This supporting file contains the results of the mixed factorial ANOVA for threshold (Table A), efficiency (Table B), unbiased hit rate (Table C), and hit rate accuracy (Table D). It also contains the hit rate accuracy for recognition of fearful facial expressions across intensities (Table E), the Shapiro-Wilk tests of normality (Table F), skewness and kurtosis of error responses to presented emotions (Table G), and a detailed error analysis summary. (DOCX) [file pone.0227019.s010.docx]

**Supporting Information**

In order to obtain the error term for our planned contrasts on signal proportion Threshold (Rosenthal & Rosnow, 1985), a 2 (Group: NSSI or Control) × 6 (Emotion: sadness, disgust, surprise, fear, anger or happiness) x 3 (50%, 75%, and 100% intensity) mixed factorial ANOVA using Threshold data was performed. Mauchly’s test indicated that the assumption of sphericity had been violated, and therefore the degrees of freedom were corrected using Greenhouse-Geisser estimates of sphericity (ε < 0.75).

**Table A. Results of mixed factorial ANOVA for threshold.**

| Source | *df* | *MS* | *F* | η^2^_p_ | *p* |
| --- | --- | --- | --- | --- | --- |
| Group | 1 | 0.003 | 0.035 | 0.001 | 0.853 |
| Intensity | 1.425 | 1.595 | 208.49 | 0.779 | 0.000 |
| Intensity x Group | 1.425 | 0.005 | 0.619 | 0.010 | 0.488 |
| Error | 84.093 | 0.008 |  |  |  |
| Emotion | 3.073 | 2.341 | 35.879 | 0.378 | 0.000 |
| Emotion x Group | 3.073 | 0.107 | 1.637 | 0.027 | 0.181 |
| Error | 181.327 | 0.065 |  |  |  |
| Intensity x Emotion | 4.456 | 0.121 | 7.16 | 0.108 | 0.000 |
| Intensity x Emotion x Group | 4.456 | 0.006 | 0.362 | 0.006 | 0.854 |
| Error | 262.915 | 0.017 |  |  |  |

*Note*. The median error term of the 9 imputed datasets is displayed in this table.

Likewise, the error term for our planned comparisons on the Efficiency data was derived from a 2 (Group: NSSI or Control) × 6 (Emotion: sadness, disgust, surprise, fear, anger or happiness) x 3 (50%, 75%, and 100% intensity) mixed factorial ANOVA. Again, Mauchly’s test indicated that the assumption of sphericity had been violated, thus the degrees of freedom were corrected using Greenhouse-Geisser estimates of sphericity (ε < 0.75).

**Table B. Results of mixed factorial ANOVA for efficiency.**

| Source | *df* | *MS* | *F* | η^2^_p_ | *p* |
| --- | --- | --- | --- | --- | --- |
| Group | 1 | 0.009 | 0.024 | 0.000 | 0.878 |
| Intensity | 1.986 | 2.595 | 36.67 | 0.383 | 0.000 |
| Intensity x Group | 1.986 | 0.103 | 1.271 | 0.021 | 0.284 |
| Error | 117.16 | 0.081 |  |  |  |
| Emotion | 4.072 | 9.736 | 22.829 | 0.279 | 0.000 |
| Emotion x Group | 4.072 | 0.257 | 0.603 | 0.010 | 0.663 |
| Error | 240.229 | 0.426 |  |  |  |
| Intensity x Emotion | 6.999 | 0.912 | 7.969 | 0.119 | 0.000 |
| Intensity x Emotion x Group | 6.999 | 0.212 | 1.849 | 0.030 | 0.077 |
| Error | 412.925 | 0.114 |  |  |  |

*Note*. The median error term of the 9 imputed datasets is displayed in this table.

The error term for the planned comparisons using the Unbiased Hit Rate (Rosenthal & Rosnow, 1985), was derived from a 2 (Group: NSSI or Control) × 6 (Emotion: sadness, disgust, surprise, fear, anger or happiness) x 3 (50% intensity, 75% intensity and 100% intensity) mixed factorial ANOVA. Mauchly’s test of sphericity indicated that the assumption of sphericity was violated, thus the degrees of freedom were once again corrected using the Greenhouse-Geisser estimates of sphericity (ε < 0.75).

**Table C. Results of mixed factorial ANOVA for unbiased hit rate.**

| Source | *df* | *MS* | *F* | η^2^_p_ | *p* |
| --- | --- | --- | --- | --- | --- |
| Group | 1 | 0.021 | 0.521 | 0.009 | 0.473 |
| Intensity | 1.451 | 0.131 | 38.629 | 0.396 | 0.000 |
| Intensity x Group | 1.451 | 0.001 | 0.341 | 0.006 | 0.642 |
| Error | 85.583 | 0.003 |  |  |  |
| Emotion | 3.987 | 0.644 | 23.991 | 0.289 | 0.000 |
| Emotion x Group | 3.987 | 0.042 | 1.578 | 0.026 | 0.181 |
| Error | 235.245 | 0.027 |  |  |  |
| Intensity x Emotion | 5.592 | 0.034 | 6.269 | 0.096 | 0.000 |
| Intensity x Emotion x Group | 5.592 | 0.003 | 0.601 | 0.718 | 0.010 |
| Error | 329.946 | 0.005 |  |  |  |

The error term for the planned comparisons using hit rate accuracy data (Rosenthal & Rosnow, 1985), was derived from a 2 (Group: NSSI or Control) × 6 (Emotion: sadness, disgust, surprise, fear, anger or happiness) x 3 (50% intensity, 75% intensity and 100% intensity) mixed factorial ANOVA. Mauchly’s test of sphericity indicated that the assumption of sphericity was violated, thus the degrees of freedom were once again corrected using the Greenhouse-Geisser estimates of sphericity (ε < 0.75).

**Table D. Results of mixed factorial ANOVA for hit rate accuracy data.**

| Source | *df* | *MS* | *F* | η^2^_p_ | *p* |
| --- | --- | --- | --- | --- | --- |
| Group |  |  |  |  |  |
| Intensity | 1.367 | 451.352 | 43.706 | 0.426 | 0.000 |
| Intensity x Group | 1.367 | 4.101 | 0.397 | 0.007 | 0.102 |
| Error | 80.680 | 10.327 |  |  |  |
| Emotion | 2.421 | 3918.857 | 23.493 | 0.285 | 0.000 |
| Emotion x Group | 2.421 | 251.602 | 1.508 | 0.025 | 0.221 |
| Error | 142.837 | 166.809 |  |  |  |
| Intensity x Emotion | 3.521 | 106.766 | 4.343 | 0.069 | 0.003 |
| Intensity x Emotion x Group | 3.521 | 22.055 | 0.897 | 0.456 | 0.456 |
| Error | 207.750 | 24.582 |  |  |  |

Results from these planned contrasts for accuracy data show that the HNSSI group were significantly less accurate at identifying fear at the 50%, 75% and 100% intensity level at the 0.05 level. No other significant results were observed for other emotions across intensity levels.

**Table E. Hit rate accuracy for recognition of fearful facial expressions across intensities.**

| *Emotion* | *Intensity* | *NSSI (Mean ± SE)* | *Control (Mean ± SE)* | *Effect Size (r)* | *p* |
| --- | --- | --- | --- | --- | --- |
| Fear | 50% | 16.433 ± 2.027 | 19.000 ± 1.994 | 0.139 | 0.044* |
|  | 75% | 18.867 ± 1.814 | 22.742 ± 1.785 | 0.207 | 0.003* |
|  | 100% | 20.067 ± 1.812 | 23.290 ± 1.783 | 0.173 | 0.012* |

* Significant at the *p* < 0.05 level

**Table F. Shapiro-Wilk tests of normality for error responses to emotions.**

| *Presented Emotion* | *Intensity* | *Error Response* | *NSSI* | | *Control* | |
| --- | --- | --- | --- | --- | --- | --- |
|  |  |  | *Statistic* | *p* | *Statistic* | *p* |
| Anger | 50 | Fear | .877 | .002 | .906 | .010 |
|  | 50 | Disgust | .947 | .140 | .812 | .000 |
|  | 50 | Happy | .935 | .067 | .904 | .009 |
|  | 50 | Sad | .958 | .276 | .892 | .005 |
|  | 50 | Surprised | .877 | .002 | .780 | .000 |
|  | 75 | Fear | .858 | .001 | .829 | .000 |
|  | 75 | Disgust | .954 | .214 | .906 | .010 |
|  | 75 | Happy | .909 | .014 | .883 | .003 |
|  | 75 | Sad | .954 | .211 | .948 | .142 |
|  | 75 | Surprised | .842 | .000 | .855 | .001 |
|  | 100 | Fear | .912 | .016 | .849 | .000 |
|  | 100 | Disgust | .892 | .005 | .862 | .001 |
|  | 100 | Happy | .914 | .019 | .941 | .088 |
|  | 100 | Sad | .935 | .067 | .938 | .074 |
|  | 100 | Surprised | .842 | .000 | .906 | .010 |
| Disgust | 50 | Fear | .784 | .000 | .860 | .001 |
|  | 50 | Anger | .835 | .000 | .863 | .001 |
|  | 50 | Happy | .819 | .000 | .862 | .001 |
|  | 50 | Sad | .795 | .000 | .838 | .000 |
|  | 50 | Surprised | .678 | .000 | .724 | .000 |
|  | 75 | Fear | .806 | .000 | .825 | .000 |
|  | 75 | Anger | .954 | .214 | .855 | .001 |
|  | 75 | Happy | .901 | .009 | .886 | .003 |
|  | 75 | Sad | .748 | .000 | .780 | .000 |
|  | 75 | Surprised | .721 | .000 | .782 | .000 |
|  | 100 | Fear | .796 | .000 | .868 | .001 |
|  | 100 | Anger | .958 | .268 | .841 | .000 |
|  | 100 | Happy | .839 | .000 | .898 | .006 |
|  | 100 | Sad | .741 | .000 | .782 | .000 |
|  | 100 | Surprised | .768 | .000 | .763 | .000 |
| Fear | 50 | Anger | .790 | .000 | .786 | .000 |
|  | 50 | Disgust | .803 | .000 | .856 | .001 |
|  | 50 | Happy | .870 | .002 | .739 | .000 |
|  | 50 | Sad | .824 | .000 | .817 | .000 |
|  | 50 | Surprised | .695 | .000 | .783 | .000 |
|  | 75 | Anger | .790 | .000 | .814 | .000 |
|  | 75 | Disgust | .666 | .000 | .847 | .000 |
|  | 75 | Happy | .849 | .001 | .840 | .000 |
|  | 75 | Sad | .846 | .001 | .790 | .000 |
|  | 75 | Surprised | .823 | .000 | .760 | .000 |
|  | 100 | Anger | .822 | .000 | .849 | .000 |
|  | 100 | Disgust | .694 | .000 | .844 | .000 |
|  | 100 | Happy | .864 | .001 | .879 | .002 |
|  | 100 | Sad | .859 | .001 | .835 | .000 |
|  | 100 | Surprised | .817 | .000 | .932 | .050 |
| Happy | 50 | Fear | .875 | .002 | .899 | .007 |
|  | 50 | Anger | .926 | .038 | .924 | .030 |
|  | 50 | Disgust | .929 | .047 | .953 | .193 |
|  | 50 | Sad | .969 | .504 | .960 | .287 |
|  | 50 | Surprised | .859 | .001 | .907 | .011 |
|  | 75 | Fear | .881 | .003 | .869 | .001 |
|  | 75 | Anger | .933 | .060 | .958 | .254 |
|  | 75 | Disgust | .958 | .270 | .921 | .026 |
|  | 75 | Sad | .947 | .144 | .936 | .063 |
|  | 75 | Surprised | .882 | .003 | .815 | .000 |
|  | 100 | Fear | .909 | .014 | .909 | .012 |
|  | 100 | Anger | .967 | .466 | .914 | .017 |
|  | 100 | Disgust | .938 | .083 | .951 | .168 |
|  | 100 | Sad | .895 | .006 | .954 | .207 |
|  | 100 | Surprised | .897 | .007 | .942 | .096 |
| Sad | 50 | Fear | .864 | .001 | .929 | .040 |
|  | 50 | Anger | .935 | .069 | .942 | .092 |
|  | 50 | Disgust | .941 | .098 | .928 | .038 |
|  | 50 | Happy | .950 | .165 | .929 | .042 |
|  | 50 | Surprised | .858 | .001 | .902 | .008 |
|  | 75 | Fear | .895 | .006 | .942 | .094 |
|  | 75 | Anger | .897 | .007 | .929 | .042 |
|  | 75 | Disgust | .917 | .022 | .930 | .045 |
|  | 75 | Happy | .946 | .129 | .936 | .064 |
|  | 75 | Surprised | .798 | .000 | .923 | .028 |
|  | 100 | Fear | .908 | .014 | .903 | .008 |
|  | 100 | Anger | .915 | .020 | .943 | .102 |
|  | 100 | Disgust | .932 | .056 | .914 | .016 |
|  | 100 | Happy | .933 | .058 | .908 | .011 |
|  | 100 | Surprised | .881 | .003 | .925 | .032 |
| Surprise | 50 | Fear | .910 | .015 | .864 | .001 |
|  | 50 | Anger | .808 | .000 | .906 | .010 |
|  | 50 | Disgust | .807 | .000 | .886 | .003 |
|  | 50 | Happy | .838 | .000 | .842 | .000 |
|  | 50 | Sad | .816 | .000 | .939 | .077 |
|  | 75 | Fear | .933 | .061 | .891 | .004 |
|  | 75 | Anger | .880 | .003 | .898 | .007 |
|  | 75 | Disgust | .843 | .000 | .882 | .003 |
|  | 75 | Happy | .860 | .001 | .896 | .006 |
|  | 75 | Sad | .820 | .000 | .920 | .023 |
|  | 100 | Fear | .922 | .030 | .899 | .007 |
|  | 100 | Anger | .933 | .060 | .898 | .006 |
|  | 100 | Disgust | .890 | .005 | .916 | .018 |
|  | 100 | Happy | .913 | .018 | .910 | .013 |
|  | 100 | Sad | .906 | .012 | .938 | .075 |

**Table G. Skewness and Kurtosis of error responses to presented emotions.**

| *Presented Emotion* | *Intensity* | *Error Response* | *NSSI* | | *Control* | |
| --- | --- | --- | --- | --- | --- | --- |
|  |  |  | *Skewness* | *Kurtosis* | *Skewness* | *Kurtosis* |
| Anger | 50 | Fear | .923 | -.052 | .459 | -.359 |
|  | 50 | Disgust | .267 | -.911 | 1.778 | 3.479 |
|  | 50 | Happy | .715 | -.150 | .627 | -.717 |
|  | 50 | Sad | -.070 | -.969 | 1.166 | 1.170 |
|  | 50 | Surprised | .258 | -.869 | 1.193 | .286 |
|  | 75 | Fear | 1.016 | .287 | 1.371 | 1.421 |
|  | 75 | Disgust | .290 | -.681 | .776 | -.228 |
|  | 75 | Happy | .847 | .149 | .450 | -1.199 |
|  | 75 | Sad | .294 | -.913 | .530 | -.304 |
|  | 75 | Surprised | .547 | -1.139 | .892 | .002 |
|  | 100 | Fear | .330 | -1.034 | .898 | -.287 |
|  | 100 | Disgust | 1.014 | 1.200 | .869 | -.398 |
|  | 100 | Happy | .754 | -.027 | .385 | -.825 |
|  | 100 | Sad | .469 | -.406 | .561 | -.633 |
|  | 100 | Surprised | 1.107 | .421 | .646 | -.446 |
| Disgust | 50 | Fear | .966 | -.339 | .887 | -.157 |
|  | 50 | Anger | 1.537 | 2.224 | 1.246 | .938 |
|  | 50 | Happy | .881 | -.414 | 1.062 | .535 |
|  | 50 | Sad | 1.190 | .167 | 1.257 | .714 |
|  | 50 | Surprised | 1.245 | .018 | 1.011 | -.493 |
|  | 75 | Fear | .776 | -.803 | .889 | -.385 |
|  | 75 | Anger | .582 | .827 | 1.257 | .950 |
|  | 75 | Happy | .738 | -.437 | .742 | -.155 |
|  | 75 | Sad | 1.321 | .277 | 1.456 | 1.118 |
|  | 75 | Surprised | 1.602 | 2.026 | 1.107 | .120 |
|  | 100 | Fear | .846 | -.706 | .746 | -.482 |
|  | 100 | Anger | .365 | -.649 | 1.202 | .648 |
|  | 100 | Happy | .819 | -.712 | .865 | .055 |
|  | 100 | Sad | 1.458 | .766 | 1.512 | 1.435 |
|  | 100 | Surprised | 1.559 | 2.513 | 1.231 | .310 |
| Fear | 50 | Anger | 1.104 | -.020 | 1.397 | 1.054 |
|  | 50 | Disgust | .895 | -.454 | 1.029 | .318 |
|  | 50 | Happy | .474 | -.931 | 1.479 | 1.341 |
|  | 50 | Sad | 1.296 | .734 | 1.370 | 1.119 |
|  | 50 | Surprised | 1.566 | 1.095 | 1.227 | .073 |
|  | 75 | Anger | .957 | -.562 | 1.101 | -.012 |
|  | 75 | Disgust | 2.067 | 3.532 | 1.094 | .539 |
|  | 75 | Happy | .819 | -.651 | 1.184 | .503 |
|  | 75 | Sad | 1.091 | .156 | 1.424 | .965 |
|  | 75 | Surprised | 1.265 | .581 | 1.801 | 2.654 |
|  | 100 | Anger | 1.386 | 1.662 | .967 | -.062 |
|  | 100 | Disgust | 1.781 | 2.098 | .774 | -.778 |
|  | 100 | Happy | .570 | -.916 | .993 | .426 |
|  | 100 | Sad | 1.044 | .298 | 1.498 | 2.243 |
|  | 100 | Surprised | 1.059 | -.221 | .105 | -1.023 |
| Happy | 50 | Fear | 1.010 | .330 | .537 | -.888 |
|  | 50 | Anger | .579 | -.723 | .586 | -.686 |
|  | 50 | Disgust | .029 | -1.290 | .356 | -.486 |
|  | 50 | Sad | .347 | -.058 | .517 | -.096 |
|  | 50 | Surprised | 1.237 | 1.381 | .293 | -1.216 |
|  | 75 | Fear | .824 | -.168 | .779 | -.592 |
|  | 75 | Anger | .763 | .529 | .400 | -.534 |
|  | 75 | Disgust | .499 | -.536 | .464 | -.518 |
|  | 75 | Sad | .538 | -.317 | .585 | -.074 |
|  | 75 | Surprised | .399 | -1.170 | 1.249 | .543 |
|  | 100 | Fear | .740 | .069 | .598 | -.510 |
|  | 100 | Anger | .258 | -.149 | .701 | -.118 |
|  | 100 | Disgust | .036 | -1.206 | .597 | .052 |
|  | 100 | Sad | .516 | -1.090 | -.172 | -.955 |
|  | 100 | Surprised | .725 | -.171 | .395 | -.567 |
| Sad | 50 | Fear | .984 | -.093 | .285 | -1.104 |
|  | 50 | Anger | .740 | .053 | .628 | -.082 |
|  | 50 | Disgust | .275 | -.763 | .915 | .953 |
|  | 50 | Happy | .577 | -.353 | .462 | -.521 |
|  | 50 | Surprised | .862 | -.188 | .315 | -1.050 |
|  | 75 | Fear | .769 | -.402 | .389 | -.368 |
|  | 75 | Anger | .933 | .102 | .400 | -.365 |
|  | 75 | Disgust | .591 | -.450 | .220 | -1.218 |
|  | 75 | Happy | .400 | -.835 | .369 | -.947 |
|  | 75 | Surprised | 1.242 | .785 | .757 | .166 |
|  | 100 | Fear | .829 | -.169 | 1.061 | .946 |
|  | 100 | Anger | .699 | -.457 | .310 | -.799 |
|  | 100 | Disgust | .109 | -.899 | .590 | -.702 |
|  | 100 | Happy | .673 | -.148 | .836 | .056 |
|  | 100 | Surprised | .892 | -.145 | .360 | -.903 |
| Surprise | 50 | Fear | .982 | .657 | 1.248 | 1.181 |
|  | 50 | Anger | .808 | -.642 | .596 | -.383 |
|  | 50 | Disgust | 1.225 | 1.077 | .604 | -.728 |
|  | 50 | Happy | 1.055 | .079 | .990 | -.086 |
|  | 50 | Sad | 1.522 | 1.827 | -.087 | -1.219 |
|  | 75 | Fear | .766 | .114 | .942 | .020 |
|  | 75 | Anger | .784 | -.370 | .605 | -.772 |
|  | 75 | Disgust | .940 | .069 | -.054 | -1.349 |
|  | 75 | Happy | .098 | -1.614 | .630 | -.832 |
|  | 75 | Sad | 1.607 | 2.680 | .765 | .769 |
|  | 100 | Fear | .761 | -.199 | .821 | -.384 |
|  | 100 | Anger | .367 | -.630 | .843 | .258 |
|  | 100 | Disgust | .280 | -.992 | .757 | .194 |
|  | 100 | Happy | .833 | -.004 | .671 | -.488 |
|  | 100 | Sad | .188 | -1.211 | .628 | -.346 |

Types of errors recognizing fear at 50%, 75% and 100%. Independent samples T-tests were conducted to assess error differences between the NSSI and control groups. No differences in error patterns were detected between the NSSI and control groups at 50% and 75% intensity. However, at 100% intensity, the NSSI group was significantly less likely to mistake fearful expressions for happy (2.1 ± 0.36) compared to the control group (3.5 ± 0.57), a statistically significant difference of -1.42 (95% CI, -2.79 to -0.04), *t*(59) = -2.062, *p* = .044, *d* = 0.53 (S2 Fig).

**Figure S1. Group differences in errors made for fear at 3 emotion intensities.**

Types of errors recognizing angry at 50%, 75% and 100%. Independent samples T-tests were conducted to assess differences in errors made identifying angry expressions between NSSI and control groups. At 50% intensity, the NSSI group was significantly less likely to mistake angry expressions for disgust (4.23 ± 0.50) compared to the control group (7.71 ± 1.40), a statistically significant difference of -3.47 (95% CI, -6.49 to -0.46), *t*(37.46) = -2.33, *p* = .025, *d* = 0.59, and were significantly more likely to mistake angry expressions for happy (4.23 ± 0.49) compared to controls (2.68 ± 0.38), a significant difference of 1.56 (95% CI, -0.14 to 2.97), *t*(49.82) = 2.20, *p* = .033, *d* = 0.57 . However, no other differences in error patterns were detected between the NSSI and control groups at 75% and 100% intensity (S3 Fig).

**Figure S2. Group differences in errors made for anger at 3 emotion intensities.**

Types of errors recognizing disgust at 50%, 75% and 100%. Independent samples T-tests were conducted to assess differences in errors made identifying disgust expressions between NSSI and control groups. At 50% intensity, the NSSI group was significantly less likely to mistake disgusted expressions for fear (1.03 ± 0.23) compared to the control group (2.06 ± 0.39), a statistically significant difference of -1.03 (95% CI, -1.93 to -0.13), *t*(38.47) = -2.31, *p* = .026, *d* = 0.59. At 100% intensity, the NSSI group was significantly more likely to mistake disgusted expressions for sad (7.47 ± 1.48) compared to controls (3.68 ± 0.76), a significant difference of 3.79 (95% CI, 0.43 to 7.15), *t*(43.39) = 2.27, *p* = .028, *d* = 0.60 . However, no other differences in error patterns were detected between the NSSI and control groups (S4 Fig).

**Figure S3. Group differences in errors made for disgust at 3 emotion intensities.**

Types of errors recognizing happy at 50%, 75% and 100%. Independent samples T-tests were conducted to assess differences in errors made identifying happy expressions between NSSI and control groups. At 75% intensity, the NSSI group was significantly less likely to mistake happy expressions for surprise (2.13 ± 0.33) compared to the control group (3.83 ± 0.55), a statistically significant difference of -1.71 (95% CI, -3.00 to -0.42), *t*(48.80) = -2.67, *p* = .010, *d* = 0.68. At 100% intensity, the NSSI group was significantly more likely to mistake happy expressions for anger (5.77 ± 0.45) compared to controls (4.32 ± 0.48), a significant difference of 1.44 (95% CI, 0.12 to 2.77), *t*(59) = 2.19, *p* = .033, *d* = 0.56. However, no other differences in error patterns were detected between the NSSI and control groups at any other intensities (S5 Fig).

**Figure S4. Group differences in errors made for happy at 3 intensities.**

Types of errors recognizing sad at 50%, 75% and 100*%.* Independent samples T-tests were conducted to assess differences in errors made identifying happy expressions between NSSI and control groups. At 50% intensity, the NSSI group was significantly less likely to mistake sad expressions for surprise (1.63 ± 0.29) compared to the control group (2.58 ± 0.26), a statistically significant difference of -0.95 (95% CI, -1.73to -0.16), *t*(59) = -2.24, *p* = .019, *d* = 0.62. At 75% intensity, the NSSI group was again significantly less likely to mistake sad expressions for surprise (1.60 ± 0.29) compared to controls (2.90 ± 0.39), a significant difference of -1.30 (95% CI, -2.27 to -0.34), *t*(59) =-2.67, *p* = .009, *d* = 0.69. However, no other differences in error patterns were detected between the NSSI and control groups at any other intensities (S6 Fig).

**Figure S5. Group differences in errors made for sad at 3 intensities.**

Types of errors recognizing surprised at 50%, 75% and 100%. Independent samples T-tests were conducted to assess differences in errors made identifying happy expressions between NSSI and control groups. At 100% intensity, the NSSI group was significantly less likely to mistake surprised expressions for sad (2.77 ± 0.39) compared to the control group (4.32 ± 0.51), a statistically significant difference of -1.56 (95% CI, -2.84 to -0.28), *t*(59) = -2.43, *p* = .018, *d* = 0.62. However, no other differences in error patterns were detected between the NSSI and control groups at any other intensities (S7 Fig).

**Figure S6. Group differences in errors made for surprise at 3 intensities.**

Types of errors for all emotions collapsed across intensities. Independent samples T-tests were conducted to assess differences in errors made when identifying the emotion expressions collapsed across intensities between NSSI and control groups. The NSSI group was significantly more likely to mistake angry expressions for the emotion of happy (4.38 ± 0.50) compared to the control group (2.98 ± 0.35), a statistically significant difference of -1.40 (95% CI, -2.61 to -0.18), *t*(59) = -2.31, *p* = .024, *d* = 0.59. The NSSI group was also significantly less likely to mistake happy (2.24 ± 0.29) and sad (1.92 ± 0.28) expression for the emotion of surprise compared to the control group (3.37 ± 0.40 and 2.81 ± 0.29), statistically significant differences of 1.12 (95% CI, -2.12 to 0.12), *t*(59) = 2.24, *p* = .029, *d* = 0.57 and 0.88 (95% CI, 1.70 to 0.07), *t*(59) = 2.18, *p* = .033, *d* = 0.56 . However, no other differences in error patterns were detected between the NSSI and control groups for any other emotions (S7-S9 Figs).

**Figure S7. Group differences in errors made for anger collapsed across intensities.**

**Figure S8. Group differences in errors made for happy collapsed across intensities.**

**Figure S9. Group differences in errors made for sad collapsed across intensities.**
